# The association of malaria morbidity with linear growth, hemoglobin, iron status, and development in young Malawian children: a prospective cohort study

**DOI:** 10.1186/s12887-018-1378-2

**Published:** 2018-12-28

**Authors:** Jaden Bendabenda, Noel Patson, Lotta Hallamaa, John Mbotwa, Charles Mangani, John Phuka, Elizabeth L. Prado, Yin Bun Cheung, Ulla Ashorn, Kathryn G. Dewey, Per Ashorn, Kenneth Maleta

**Affiliations:** 1 College of Medicine, Department of Public Health, School of Public Health, University of Malawi, Mahatma Gandhi Road, Private Bag 360, Blantyre 3, Malawi; 2 Faculty of Medicine and Life Sciences, Center for Child Health Research, University of Tampere, Tampere, Finland; 3 Program in Health Services and Systems Research and Centre for Quantitative Medicine, Duke-National University of Singapore Graduate Medical School, Singapore, Singapore; 4 Department of Nutrition, University of California, Davis, Davis, CA USA; 5 School of Public Health, University of the Witwatersrand, Johannesburg, South Africa; 6 Department of Applied Studies, Malawi University of Science and Technology, Thyolo, Malawi; 7 Leeds Institute for Data Analytics, University of Leeds, Leeds, UK

**Keywords:** Children, Growth faltering, Malaria, Morbidity, Infections, Stunting, iLiNS studies, Longitudinal studies

## Abstract

**Background:**

Although poor complementary feeding is associated with poor child growth, nutrition interventions only have modest impact on child growth, due to high burden of infections. We aimed to assess the association of malaria with linear growth, hemoglobin, iron status, and development in children aged 6–18 months in a setting of high malaria and undernutrition prevalence.

**Methods:**

Prospective cohort study, conducted in Mangochi district, Malawi. We enrolled six-months-old infants and collected weekly data for ‘presumed’ malaria, diarrhea, and acute respiratory infections (ARI) until age 18 months. Change in length-for-age z-scores (LAZ), stunting, hemoglobin, iron status, and development were assessed at age 18 months. We used ordinary least squares regression for continuous outcomes and modified Poisson regression for categorical outcomes.

**Results:**

Of the 2723 children enrolled, 2016 (74.0%) had complete measurements. The mean (standard deviation) incidences of ‘presumed’ malaria, diarrhea, and ARI, respectively were: 1.4 (2.0), 4.6 (10.1), and 8.3 (5.0) episodes/child year. Prevalence of stunting increased from 27.4 to 41.5% from 6 to 18 months. ‘Presumed’ malaria incidence was associated with higher risk of stunting (risk ratio [RR] = 1.04, 95% confidence interval [CI] = 1.01 to 1.07, *p* = 0.023), anemia (RR = 1.02, 95%CI = 1.00 to 1.04, *p* = 0.014) and better socio-emotional scores (B = − 0.21, 95%CI = − 0.39 to − 0.03, *p* = 0.041), but not with change in LAZ, haemoglobin, iron status or other developmental outcomes. Diarrhea incidence was associated with change in LAZ (B = − 0.02; 95% CI = − 0.03 to − 0.01; *p* = 0.009), stunting (RR = 1.02; 95% CI = 1.01 to 1.03; *p* = 0.005), and slower motor development. ARI incidence was not associated with any outcome except for poorer socio-emotional scores.

**Conclusion:**

In this population of young children living in a malaria-endemic setting, with active surveillance and treatment, ‘presumed’ malaria is not associated with change in LAZ, hemoglobin, or iron status, but could be associated with stunting and anemia. Diarrhea was more consistently associated with growth than was malaria or ARI. The findings may be different in contexts where active malaria surveillance and treatment is not provided.

**Trial registration:**

NCT00945698 (July 24, 2009) and NCT01239693 (November 11, 2010).

## Introduction

Although poor complementary feeding is associated with poor child growth, many interventions designed to improve complementary foods only have modest impact on growth [1], possibly due to a high burden of infections in children [2, 3]. Studies in which morbidity treatment was integrated with a complementary feeding intervention demonstrated improved linear growth [4] and developmental outcomes in children [5, 6], suggesting the importance of reducing the burden of infections along with improved diet to promote child growth and development.

Longitudinal studies have reported a significant inverse association of diarrhea with growth [7–9]. However, studies on the association of malaria with growth and development have either reported inconsistent results or had cross-sectional designs, which makes it difficult to assess causality or directionality of association [10–13]. This has prevented the inclusion of malaria as a determinant of stunting in the Lives Saved Tool (LiST) model [14].

The International Lipid-based Nutrient Supplements (iLiNS) Project DOSE and DYAD-M studies were randomized controlled trials conducted in Malawi to study the impact of lipid-based nutrient supplements (LNS) on growth of children [15, 16]. The aim of this analysis was to assess the association of malaria with linear growth, hemoglobin, iron status, and child development. Our hypothesis was that linear growth, hemoglobin, iron status, and developmental outcomes at age 18 mo would be poorer in children with higher incidence of malaria from age 6 to 18 mo. We also analyzed the association of diarrhea and acute respiratory infections (ARI) with linear growth, hemoglobin, iron status, and developmental outcomes.

## Methods

### Study setting

The iLiNS-DOSE and iLiNS-DYAD-M studies were conducted in one public district hospital (Mangochi), one mission hospital (St Martins), and two rural public health centers (Lungwena and Namwera) in Mangochi District, Southern Malawi. The total catchment population of 180,000 largely subsisted on farming and fishing. In Malawian children aged < 5 years, the prevalence of reported fever (a proxy for malaria), diarrhea and ARI was 29, 22 and 5%, respectively, with seasonal fluctuations [17]. Malaria is endemic in Malawi and the study area has high malaria transmission with high temperature and frequent rainfall from October through April [18].

### Study design and data collection

In the iLiNS-DOSE study, 6-mo old children were randomly allocated to one of five intervention groups provided with different doses or formulations of LNS or to a control group that did not receive LNS during the 12-mo study period, between November 2009 and May 2012. In the iLiNS-DYAD-M study, pregnant women < 20 weeks’ gestation were randomly allocated to one of three groups to receive iron and folic acid (IFA), multiple micronutrients (MMN) or a small-quantity (20 g) of LNS daily. After delivery, women in the IFA group received placebo tablets, while MMN and LNS supplementation was continued up to 6 mo postpartum. Children of mothers in the LNS group also received LNS 10 g twice daily from age 6 to 18 mo. This study was conducted from February 2011 to April 2015. Details of study design, randomization and enrolment for the two studies were explained in the main outcome papers [15, 16].

In both studies, research assistants visited the children’s homes every week from age 6 to 18 mo to interview the guardians about the child’s health in the previous 7 days using a structured questionnaire. The information was complemented by a picture calendar filled out by the guardians daily to aid memory of their children’s morbidity status. The use of maternal interviews as a means of collecting data on child morbidity has been validated in previous studies [19, 20]. The research assistants referred all cases of ‘presumed’ malaria (presence of fever) to the nearby health facility for treatment with lumefantrine/artemether, the nationally recommended antimalarial drug. The children were followed throughout the year, covering periods of both high and low malaria transmission.

Anthropometric measurements were taken at age 6 mo and 18 mo. Study anthropometrists measured the infant’s length with a high-quality length board (Harpenden Infantometer; Holtain Limited) and recorded it to the nearest 1 mm. They weighed unclothed infants with electronic infant weighing scale (SECA 735; Seca GmbH & Co), recording to the nearest 10 g. The anthropometrists were trained and their measurement reliability was verified at the start of the study and at 6-mo intervals thereafter with methods adapted from the procedures used in the WHO Multicentre Growth Reference Study [21]. The anthropometrists calibrated all equipment with standard weights and length rods daily.

We assessed iron status at age 6 mo and 18 mo by measuring the zinc protoporphyrin (ZPP) concentration in unwashed venous blood sample using a hematofluorometer (206D, AVIV Biomedical Inc., Lakewood, NJ, USA). About 5–7 ml of blood was collected by venepuncture using a 23-gauge needle into 7.5 ml evacuated, trace element-free polyethylene tubes containing lithium heparin (Sarstedt AG & Co, Nümbrecht, Germany). The samples were kept covered in aluminium and away from light, in a refrigerator or on ice, and processed within 2 h of collection. We measured blood hemoglobin (Hb) concentration at age 6 mo and 18 mo from a drop of blood taken from a finger prick and collected in a microcuvette. Hb analysis was conducted on-site using a Hemo-Cue instrument (Hemocue 201+, HemoCue® AB, Ängelholm, Sweden).

We assessed fine and gross motor development at age 18 mo using the Kilifi Developmental Inventory (KDI) developed in Kenya [22]. Language development was assessed using a 100-word vocabulary checklist by maternal interview based on the MacArthur-Bates Communicative Development Inventory [23] adapted for the local languages, and 18-mo socio-emotional development was assessed using the Profile of Social and Emotional Development (PSED), also developed in Kenya. The child’s mood during the KDI assessment was rated as positive (smiling/laughing) or not positive (crying/inconsolable, changeable/mood swings, or no visible emotions). The child’s interaction with the assessor during the KDI was rated as positive (friendly) or not positive (avoidant and withdrawn, clings to family member, hesitant/when approached will accept reluctantly, difficult to engage in tasks, or inappropriate approaches to the assessor). The child’s activity level during the KDI was rated as positive (active and maintains interest) or not positive (unarousable, sleepy and can hardly be awakened, sleepy but easily awakened, does not spontaneously engage in activity, and awake but loses interest). The KDI, vocabulary, and PSED scores showed high inter-rater agreement and moderate to high test-retest reliability in this study setting [24, 25].

### Definition of the predictors and the outcomes

We used a presumptive diagnosis of malaria derived from episodes of fever during the previous week, reported by the guardians. To ensure the diagnoses were mutually exclusive, we created an algorithm whereby any fever with a diarrhea episode (three or more loose stools in 24 h) was categorized as diarrhea; any fever in the presence of any respiratory symptoms (cough, rapid or difficult breathing and nasal discharge) was categorized as ARI. ‘Presumed’ malaria was defined as any fever episode in the absence of diarrhea and respiratory symptoms.

An episode of ‘presumed’ malaria, ARI or diarrhea was defined as the period starting from the day the child had the symptoms when preceded by at least 2 days of no symptoms or no data. The episode ended on the last day the child had the symptoms which was then followed by at least 2 symptom-free days. Incidence of ‘presumed’ malaria, ARI or diarrhea for each child from age 6 to 18 mo was calculated as total episodes / total follow up years at risk.

Longitudinal prevalences of common childhood symptoms (fever, diarrhea, and cough) from age 6 to 18 mo were defined as the number of days with the symptom divided by the total number of days of observation for each child [26].

We calculated age- and sex-standardized anthropometric indices [length-for-age z score (LAZ), weight-for-age z score (WAZ), and weight-for-length z score (WLZ)] based on the WHO Child Growth Standards [21] and considered values below – 2.0 indicative of underweight, stunting and wasting, respectively. Change in LAZ for each child was calculated as the difference between LAZ at age 18 mo and LAZ at age 6 mo.

Iron deficiency at age 6 mo and 18 mo was defined as whole blood ZPP > 70 μmol/mole heme [27]. Anemia at age 6 mo was defined as blood Hb concentration < 105 g/L [28] while anemia at age 18 mo was defined as blood Hb concentration < 110 g/L [29].

From the child development data at age 18 mo, fine motor scores were calculated as the sum of 34 KDI fine motor items, each scored 0 or 1, gross motor scores were calculated as the sum of 35 KDI gross motor items, each scored 0 or 1 [22] and vocabulary score was the maternal-reported child expressive vocabulary out of the 100-word checklist. For these outcomes, moderate to severe delay was defined as the bottom 25% of the sample. The socio-emotional score was calculated as the sum of 19 PSED items. Moderate to severe delay was defined as the top 25% of our sample (a higher score indicates less advanced socio-emotional development).

### Statistical analysis

We included in the analysis children who had outcomes measured at age 18 mo. For all continuous outcomes, we used ordinary least squares regression to assess the association between malaria incidence and each outcome; and for all binary outcomes, we used modified Poisson regression (with a robust variance estimator) [30].

We first assessed whether the relationship between the predictor and each outcome differed between the two studies. However, the interaction term was not statistically significant indicating that this relationship was not different between the two studies therefore we pooled data from the two cohorts. We then constructed multivariate models to determine which variables independently predicted the outcomes. We included all theoretically relevant variables, regardless of whether they were statistically significant or not after the bivariate analysis. The following variables collected at age 6 mo were included in the models: child sex, LAZ, WLZ, Hb, iron status, maternal education and household food insecurity access (HFIA) score generated by summing the value of responses to nine questions regarding food insecurity [31]. We also included in the models, from age 6 to 18 mo, the incidence of diarrhea and ARI, and whether the child received an intervention (LNS) or not. For the risk of stunting at age 18 mo, we included in the model stunting at age 6 mo (in place of LAZ). In addition, all developmental outcomes were adjusted for the child’s mood, activity level, age and interaction with the assessor.

We assessed collinearity among the variables (e.g. LAZ vs WLZ at age 6 mo). If the variables were highly collinear (> 0.5), we dropped the one that was less strongly associated with the outcomes. We accounted for intracluster correlation due to twins using generalised estimating equations [32].

We also performed exploratory analyses by using frequency of malaria episodes (from age 6 to 18 mo) as a categorical variable (no episode, one episode, and > 1 episodes groups). In addition, we conducted stratified analyses by stunting at age 6 mo. Although we performed bivariate analyses for each individual variable, we will only report the results from multivariate analysis.

We used Stata version 14 (StataCorp, Texas, USA) for all the analyses.

## Results

### Baseline characteristics and descriptive statistics

**Fig. 1:**
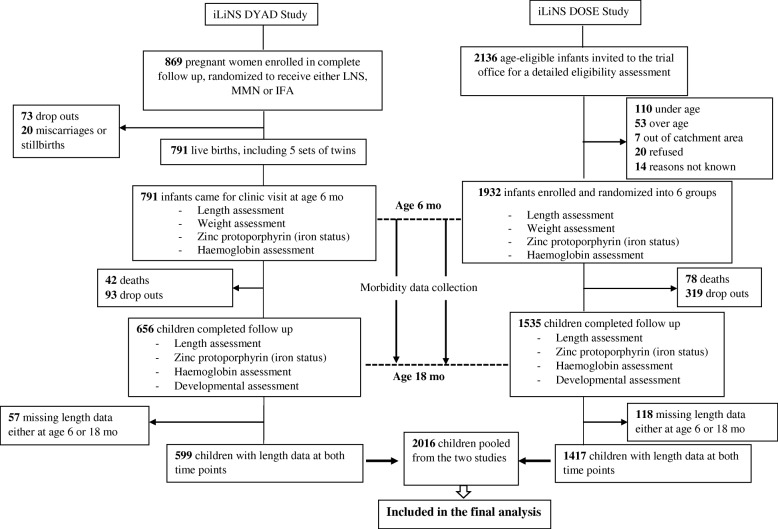
Flow chart of the children enrolled and included in the final analysis. The figure shows the number of children enrolled, children lost to follow up, and children who were eventually included in the study from the iLiNS DOSE and iLiNS DYAD-M cohorts

Of the 2723 children enrolled in the two study cohorts, 2016 (74.0%) had length measured both at age 6 mo and 18 mo (1417 children from the iLiNS DOSE study and 599 children from the iLiNS DYAD-M study). These were included in the final analysis (Fig. 1). The characteristics of these children at age 6 mo are summarized in Table 1.

**Table 1:** Participant characteristics at age 6 mo

Variable	DOSE^a^	DYAD-M^a^	Pooled data^a^
Number of children^b^	1417	599	2016
Proportion of boys	50.5%	47.6%	49.6%
Mean (SD) weight, kg	7.0 (1.0)	7.3 (1.0)	7.1 (1.0)
Mean (SD) length, cm	63.4 (2.4)	64.2 (2.6)	63.6 (2.5)
Mean (SD) weight-for-age z-score	−0.7 (1.1)	− 0.5 (1.2)	− 0.7 (1.2)
Mean (SD) length-for-age z-score	−1.4 (1.0)	− 1.2 (1.1)	− 1.4 (1.1)
Mean (SD) weight-for-length z-score	0.3 (1.1)	0.4 (1.1)	0.3 (1.1)
Mean (SD) hemoglobin, g/L	103.6 (16.1)	103.7 (15.3)	103.6 (15.9)
Proportion with LAZ < −2 scores	29.1%	23.4%	27.4%
Proportion with Hb < 105 g/L	50.6%	54.4%	51.7%
Proportion with ZPP > 70 μmole/mole heme^c^	68.7%	69.8%	69.0%
Proportion with malaria^d^	16.7%	9.7%	14.5%
Mean (SD) maternal education, completed years	4.7 (3.6)	3.8 (3.5)	4.4 (3.6)
Mean (SD) maternal age, years	26.1 (6.2)	25.1 (6.0)	25.8 (6.1)
Mean (SD) Household Food Insecurity Access Score	6.5 (6.0)	5.0 (4.3)	6.0 (5.6)

^a^Values are n, mean (SD) or proportions

^b^Children who had length data at age 6 mo and 18 mo

^c^Measured from unwashed venous blood

^d^Measured by malaria antigen Rapid Diagnosis Test (mRDT)

The 2016 children included in the final analysis contributed 1647.9 child years of follow up, i.e. the mean (standard deviation) [SD] duration of follow up was 298 (61) days / child. A total of 24,024 morbidity episodes were reported during the home visits. Of these, 9.7% (2324/24024) were episodes of ‘presumed’ malaria. The rest of the morbidity episodes were due to: acute respiratory infections (ARI), 55.6% (13,360/24024); diarrhea, 33.6% (8083/24024); and minor conditions, 1.1% (257/24024).

Overall, the mean (SD) incidence of all illnesses combined was 14.8 (6.8) episodes per child year. The mean (SD) incidence of ‘presumed’ malaria was 1.4 (2.0) episodes per child year. The mean (SD) incidence of ARI was 8.3 (5.0) episodes per child year and the mean (SD) incidence of diarrhea was 4.6 (10.1) episodes per child year. The longitudinal prevalences of common childhood symptoms (fever, diarrhea, and cough) from age 6 to 18 mo were: 7.5%; 3.4%; and 11.7%, respectively.

During the 12-mo follow up period, 39.0% (787/2016) of the children did not report any episode of ‘presumed’ malaria, 30.3% (611/2016) reported one episode and 30.7% (618/2016) reported > 1 malaria episodes. The children who reported > 1 malaria episodes were responsible for 73.7% (1713/2324) of all ‘presumed’ malaria episodes reported in the two studies.

At age 18 mo, the mean (SD) length-for-age z-scores (LAZ), weight-for-age z-scores (WAZ) and weight-for-length (WLZ) scores were − 1.8 (1.1), − 1.0 (1.1) and − 0.2 (1.1) respectively. The proportions of children who were stunted, underweight and wasted were 41.5, 16.6 and 5.0%, respectively. The median (25th, 75th centile) zinc protoporphyrin (ZPP) concentration was 74 (51, 114) μmole/mole heme and the proportion with iron deficiency was 54.1%. The mean (SD) hemogobin (Hb) concentration was 108.5 (15.1) g/L and the proportion with anemia was 50.5%. The mean (SD) scores for fine motor, gross motor, language and Profile of Social and Emotional Development (PSED) were 20.9 (2.2), 17.3 (2.6), 26.2 (5.0) and 16.2 (5.4) respectively.

### Association of malaria with linear growth

The mean (SD) change in LAZ from age 6 to 18 mo was − 0.44 (0.77). In multivariate analysis, there was no association between the incidence of ‘presumed’ malaria and change in LAZ from age 6 to 18 mo, adjusted for LAZ at age 6 mo (B = − 0.02, 95% CI = − 0.04 to 0.01, *p* = 0.069) (Table 2).

**Table 2:** Association of infectious disease morbidity from age 6 to 18 mo with change in LAZ and stunting at age 18 mo

Variables	Mean change in LAZ from age 6 to 18 mo		Stunting at age 18 mo	
	(*N* = 2016)		(*N* = 2016)	
	Regression coefficient^a^ (95% CI)	*P*-value	Risk ratio^b^ (95% CI)	*P*-value
Incidence^c^ of ‘presumed’ malaria	− 0.02 (− 0.04 to 0.01)	0.069	1.04 (1.01 to 1.07)	0.023
> 1 malaria episodes (vs no malaria episode)	− 0.04 (− 0.12 to 0.04)	0.363	1.23 (1.02 to 1.49)	0.034
Incidence^c^ of diarrhea	− 0.02 (− 0.03 to − 0.01)	0.009	1.02 (1.01 to 1.03)	0.005
Incidence^c^ of ARI	0.01 (− 0.01 to 0.01)	0.614	0.99 (0.98 to 1.00)	0.097
Other predictors^d^:				
Female sex (vs. male)	0.09 (0.02 to 0.16)	0.012	0.92 (0.78 to 1.09)	0.352
LAZ at age 6 mo	−0.27 (− 0.30 to − 0.24)	< 0.001	Not included in the model	Not included in the model
WLZ at age 6 mo	0.14 (0.11 to 0.17)	< 0.001	0.76 (0.69 to 0.82)	< 0.001
Hemoglobin (g/L) at age 6 mo	0.01 (−0.01 to 0.01)	0.943	0.98 (0.97 to 0.99)	0.009
Stunting at age 6 mo	Not included in the model	Not included in the model	3.02 (2.72 to 3.35)	< 0.001
HFIA score	−0.01 (−0.01 to 0.01)	0.155	1.02 (1.00 to 1.03)	0.025

*ARI* acute respiratory infection, *CI* confidence interval, *HFIA* household food insecurity access, *LAZ* length for age z-score, *WLZ* weight for length z-score

^a^Obtained by ordinary least squares regression

^b^Obtained by modified poisson regression (with a robust variance estimator)

^c^Total episodes/child years at risk

^d^Only predictors that showed statistical significance in any of the multivariate models are presented. Other variables entered in the regression, but not significant in any model, were: iron status at age 6 mo; maternal education; and whether the child received an intervention (LNS) during the study period

The proportion of children who were stunted increased from 27.4% at age 6 mo to 41.5% at age 18 mo. In multivariate analysis, the incidence of ‘presumed’ malaria was associated with higher risk of stunting at age 18 mo, adjusted for stunting at age 6 mo (RR = 1.04, 95% CI = 1.01 to 1.07, *p* = 0.023) (Table 2). When categorized by frequency of malaria episodes and adjusted for stunting at age 6 mo, children with > 1 malaria episodes from age 6 to 18 mo had higher risk of stunting at age 18 mo compared to children with zero malaria episodes (RR = 1.39, 95% CI = 1.13 to 1.70, *p* = 0.002).

### Association of malaria with hemoglobin, anemia and iron status

The incidence of ‘presumed’ malaria from age 6 to 18 mo was associated with higher risk of anemia at age 18 mo, adjusted for hemoglobin at age 6 mo (RR = − 0.12; 95% CI = − 0.20 to − 0.04; *p* = 0.002) but not with hemoglobin or iron deficiency at age 18 mo (Table 3).

**Table 3:** Association of infectious disease morbidity from age 6 to 18 mo with hemoglobin, anemia and iron deficiency at age 18 mo

Variables	Mean hemoglobin (g/L) at age 18 mo		Anemia^a^ at age 18 mo		Iron deficiency^b^ at age 18 mo	
	(*N* = 1157)		(*N* = 1157)		(*N* = 1707)	
	Regression coefficient^c^	*P*-value	Risk ratio^d^	*P*-value	Risk ratio^d^	*P*-value
	(95% CI)		(95% CI)		(95% CI)	
Incidence^e^ of ‘presumed’ malaria	−0.43 (− 1.21 to 0.34)	0.273	1.02 (1.00 to 1.04)	0.014	1.01 (0.99 to 1.02)	0.223
> 1 malaria episodes (vs no malaria episode)	−1.80 (−3.74 to 0.14)	0.068	1.14 (1.02 to 1.27)	0.022	1.05 (0.95 to 1.17)	0.304
Incidence^e^ of diarrhea	0.01 (−0.06 to 0.08)	0.767	1.00 (0.99 to 1.01)	0.370	1.00 (0.99 to 1.01)	0.776
Incidence^e^ of ARI	0.09 (− 0.11 to 0.31)	0.392	1.00 (0.99 to 1.01)	0.671	1.00 (0.99 to 1.01)	0.772
Other predictors^f^:						
WLZ at age 6 mo	1.10 (0.21 to 1.99)	0.015	0.98 (0.94 to 1.02)	0.248	0.99 (0.96 to 1.03)	0.811
Hemoglobin (g/L) at age 6 mo	0.21 (0.15 to 0.28)	< 0.001	0.98 (0.97 to 0.99)	< 0.001	1.05 (0.96 to 1.14)	0.283
ZPP > 70 μmole/mole heme at age 6 mo	−0.64 (− 2.42 to 1.14)	0.483	1.03 (0.92 to 1.15)	0.632	1.95 (1.71 to 2.23)	< 0.001

*ARI* acute respiratory infection, *CI* confidence interval, *WLZ* weight for length z-score, *ZPP* zinc protoporphyrin

^a^Defined as blood hemoglobin concentration < 110 g/L [29]

^b^Defined as whole blood ZPP > 70 μmol/mole heme [27]

^c^Obtained by ordinary least squares regression

^d^Obtained by modified poisson regression (with a robust variance estimator)

^e^Total episodes/child years at risk

^f^Only predictors that showed statistical significance in any of the multivariate models are presented. Other variables entered in the regression, but not significant in any model, were: length for age z-score at age 6 mo; child sex; maternal education; household food insecurity access score; and whether the child received an intervention (lipid-based nutrient supplements) during the study period

### Association of malaria with child development

The association of incidence of ‘presumed’ malaria from age 6 to 18 mo with child development was significant for PSED scores (B = − 0.21; 95% CI = 0.39 to − 0.03; *p* = 0.041), but not for the other domains of child development, adjusted for the covariates listed in the footnotes to Tables 4 and 5**.**

**Table 4:** Association of infectious disease morbidity from age 6 to 18 mo with developmental scores at age 18 mo

Variables	Mean developmental scores at age 18 mo							
	(*N* = 2016)							
	Fine motor scores		Gross motor scores		Language scores		PSED scores	
	Regression coefficient^a^	*P*-value	Regression coefficient^a^	*P*-value	Regression coefficient^a^	*P*-value	Regression coefficient^a^	*P*-value
	(95% CI)		(95% CI)		(95% CI)		(95% CI)	
Incidence^b^ of ‘presumed’ malaria	−0.06 (− 0.13 to 0.01)	0.117	0.07 (− 0.17 to 0.01)	0.070	−0.01 (− 0.09 to 0.06)	0.692	−0.21 (− 0.39 to − 0.03)	0.041
> 1 malaria episodes (vs no malaria episode)	−0.09 (− 0.34 to 0.16)	0.488	−0.17 (− 0.45 to 0.12)	0.250	−0.09 (− 0.35 to 0.16)	0.460	−0.48 (− 1.11 to 0.15)	0.132
Incidence^b^ of diarrhea	−0.06 (− 0.11 to − 0.01)	0.050	−0.02 (− 0.03 to − 0.01)	< 0.001	0.01 (− 0.05 to 0.05)	0.984	−0.02 (− 0.04 to 0.01)	0.126
Incidence^b^ of ARI	0.01 (− 0.02 to 0.04)	0.481	0.03 (−0.01 to 0.05)	0.067	0.01 (−0.02 to 0.04)	0.432	0.08 (0.03 to 0.14)	0.004
Other predictors^c^								
Female sex (vs. male)	−0.27 (− 0.54 to − 0.01)	0.043	−0.67 (− 0.94 to − 0.39)	< 0.001	0.13 (− 0.13 to 0.39)	0.315	− 1.28 (− 1.92 to − 0.64)	< 0.001
LAZ at age 6 mo	0.20 (0.07 to 0.33)	0.002	0.32 (0.18 to 0.45)	< 0.001	0.28 (0.15 to 0.41)	< 0.001	0.16 (− 0.48 to 0.16)	0.322
HFIA score	0.04 (0.02 to 0.06)	0.001	0.01 (−0.01 to 0.03)	0.392	−0.02 (− 0.04 to − 0.01)	0.044	0.01 (− 0.04 to 0.06)	0.719
Child’s mood	0.95 (0.70 to 1.19)	< 0.001	0.92 (0.64 to 1.19)	< 0.001	−0.20 (− 0.44 to 0.04)	0.108	− 0.75 (−1.36 to −.14)	0.016
Activity level	0.48 (0.10 to 0.88)	0.014	0.46 (0.02 to 0.90)	0.041	0.39 (−0.01 to 0.78)	0.051	0.94 (−0.04 to 1.92)	0.061

*ARI* acute respiratory infection, *CI* confidence interval, *HFIA* household food insecurity access, *LAZ* length for age z-score, *PSED* Profile of Social and Emotional Development

^a^Obtained by ordinary least squares regression

^b^Total episodes/child years at risk

^c^Only predictors that showed statistical significance in any of the multivariate models are presented. Other variables entered in the regression, but not significant in any model, were: weight for length z-scores at age 6 mo; hemoglobin concentration; iron status; maternal education; interaction with the assessor during the Kilifi Developmental Inventory (KDI) assessment; and whether the child received an intervention (lipid-based nutrient supplements) during the study period

**Table 5:** Association of infectious disease morbidity from 6 to 18 mo with developmental delay at 18 mo

Independent Variables	Developmental delay^a^							
	(*N* = 2016)							
	Fine motor delay		Gross motor delay		Language delay		PSED delay	
	Risk ratio^b^	*P*-value	Risk ratio^b^	*P*-value	Risk ratio^b^	*P*-value	Risk ratio^b^	*P*-value
	(95% CI)		(95% CI)		(95% CI)		(95% CI)	
Incidence^c^ of ‘presumed’ malaria	1.03 (0.96 to 1.09)	0.435	1.04 (1.00 to 1.09)	0.059	0.99 (0.94 to 1.06)	0.926	0.95 (0.88 to 1.02)	0.145
> 1 malaria episodes (vs no malaria episode)	0.96 (0.78 to 1.19)	0.723	1.06 (0.89 to 1.27)	0.508	1.00 (0.82 to 1.23)	0.971	0.82 (0.66 to 1.03)	0.094
Incidence^c^ of diarrhea	1.01 (1.00 to 1.02)	0.011	1.01 (1.00 to 1.02)	< 0.001	1.00 (0.99 to 1.01)	0.129	1.00 (0.99 to 1.01)	0.953
Incidence^c^ of ARI	0.99 (0.97 to 1.01)	0.367	0.99 (0.98 to 1.01)	0.445	0.99 (0.97 to 1.01)	0.338	1.02 (1.00 to 1.04)	0.025
Other predictors^d^								
Female sex (vs. male)	1.13 (0.91 to 1.41)	0.253	1.50 (1.22 to 1.85)	< 0.001	0.92 (0.74 to 1.14)	0.438	0.77 (0.61 to 0.96)	0.023
LAZ at age 6 mo	0.90 (0.81 to 1.00)	0.059	0.82 (0.73 to 0.91)	< 0.001	0.87 (0.78 to 0.97)	0.014	0.97 (0.86 to 1.08)	0.533
WLZ at age 6 mo	0.84 (0.76 to 0.93)	0.001	0.97 (0.88 to 1.07)	0.517	0.97 (0.88 to 1.07)	0.542	0.92 (0.83 to 1.03)	0.145
HFIA score	0.98 (0.96 to 0.99)	0.034	0.99 (0.98 to 1.01)	0.702	1.01 (0.99 to 1.03)	0.091	1.02 (0.99 to 1.04)	0.075
Child’s mood	0.38 (0.29 to 0.49)	< 0.001	0.67 (0.55 to 0.83)	< 0.001	1.08 (0.89 to 1.33)	0.435	0.78 (0.63 to 0.97)	0.023
Activity level	0.92 (0.67 to 1.25)	0.587	0.67 (0.51 to 0.87)	0.003	0.91 (0.65 to 1.29)	0.610	1.57 (1.12 to 2.21)	0.009

*ARI* acute respiratory infection, *CI* confidence interval, *HFIA* household food insecurity access, *LAZ* length for age z-score, *PSED* Profile of Social and Emotional Development

^a^Defined as the bottom 25% of our sample

^b^Obtained by modified poisson regression (with a robust variance estimator)

^c^Total episodes/child years at risk

^d^Only predictors that showed statistical significance in any of the multivariate models are presented. Other variables entered in the regression, but not significant in any model, were: hemoglobin concentration; iron status; maternal education; interaction with the assessor during the Kilifi Developmental Inventory (KDI) assessment; and whether the child received an intervention (lipid-based nutrient supplements) during the study period

### Association of diarrhea and ARI with linear growth, hemoglobin, iron status, and developmental outcomes

In multivariate analysis, incidence of diarrhea from age 6 to 18 mo was associated with change in LAZ from age 6 to 18 mo (B = − 0.02; 95% CI = − 0.03 to − 0.01; *p* = 0.009), higher risk of stunting at age 18 mo (RR = 1.02; 95% CI = 1.01 to 1.03; *p* = 0.005) (Table 2), lower gross motor scores at age 18 mo (B = − 0.02; 95% CI = − 0.03 to − 0.01; *p* < 0.001), and higher risk of gross motor delay (RR = 1.01; 95% CI = 1.00 to 1.02; *p* < 0.001) and fine motor delay (RR = 1.01; 95% CI = 1.00 to 1.02; *p* = 0.011) at age 18 mo (Tables 4 and 5).

The incidence of ARI from age 6 to 18 mo was not significantly associated with growth or other outcomes except for PSED scores (B = 0.08; 95% CI = 0.03 to 0.14; *p* = 0.004), and PSED delay (RR = 1.02; 95% CI = 1.00 to 1.04; *p* = 0.025) (Tables 4 and 5).

## Discussion

We tested the hypothesis that the linear growth, hemoglobin, iron status, and developmental outcomes at age 18 mo would be poorer in children with higher incidence of ‘presumed’ malaria. In a sample of 2016 Malawian children aged 6–18 mo, we found that malaria was not associated with change in LAZ, fine motor scores, gross motor scores, language development, iron status or hemoglobin concentration. Higher incidence of ‘presumed’ malaria was associated with higher risk of stunting and anemia (i.e: one additional episode of ‘presumed’ malaria per year was associated with 4 and 2% higher risk of stunting and anemia, respectively). Higher incidence of ‘presumed’ malaria was also associated with lower socio-emotional scores (i.e: one additional episode of ‘presumed’ malaria per year was associated with a reduction in PSED scores by 0.21), suggesting that children with higher malaria incidence tended to have fewer socio-emotional problems, possibly because malaria causes lethargy and inactivity which may manifest as fewer behavioral problems.

Our study had several strengths: the weekly home morbidity data collection for 1 year provided comprehensive data covering periods of both high and low malaria transmission; the longitudinal design made it possible to correlate the malaria exposure with the outcomes and interpret the directionality of association; and pooling of data from two studies helped us draw conclusions from a large sample. Although we did not calculate post-hoc power for this analysis, with a large sample size of 2016 children and narrow confidence intervals obtained for most of the morbidity outcomes, we believe the study was powered to detect clinically meaningful associations.

Our results should be interpreted with caution because we excluded children who did not have the outcomes measured at age 18 mo (26% of the sample), resulting in possible survival bias. However, this attrition rate is similar to that of other studies with a long follow up period [9, 10]. Moreover, we expect that the children lost to follow up may have had worse outcomes, which probably would have increased the strength of association in our findings.

Another possible cause of bias is the presumptive diagnosis of malaria, which could lead to misclassification. There is overlap in the symptoms of malaria, diarrhea and ARI [33] which may affect the sensitivity and specificity of presumptive malaria diagnosis depending on the intensity of malaria transmission. For example, presumptive malaria diagnosis usually has higher sensitivity and lower specificity in areas of high malaria transmission compared to areas of low transmission [34, 35]. With the global decline in malaria incidence and availability of malaria Rapid Diagnostic Tests (mRDTs), the WHO in 2010 recommended antimalarial treatment be provided when there is evidence of a positive malaria test result [36]. However, at the time of conducting our study, mRDTs had not been rolled out nationwide, hence presumptive malaria diagnosis was used not only in this study but also in national prevalence surveys [18, 37], according to the practice of Integrated Management of Childhood Illness (IMCI) [38, 39]. Furthermore, in exploratory analysis using hospital diagnosed malaria (confirmed by mRDT, albeit with a lot of missing data), the direction of the associations was similar, suggesting that our findings are still valid (data not shown).

Our research assistants referred the children suspected of malaria to a clinic for treatment; the active surveillance and early treatment may have helped improve the study outcomes, which may have resulted in underestimation of the associations.

It is also possible that the association of malaria with stunting, anemia, and PSED scores was significant by chance due to multiple testing [40]. However, we believe the chance finding was less likely for the significant associations of diarrhoea and ARI with the outcomes because these associations were relatively strong based on *p*-values.

The available evidence on the association of malaria with growth and other outcomes is inconclusive. Some studies have reported significant associations of malaria with stunting, hemoglobin concentration, iron status and child development [11, 12, 41–44]. Children living in settings where infectious diseases are frequent and complementary food is of poor quality often fail to achieve catch up growth after illness episodes [45], hence frequent malaria could be associated with growth faltering. Earlier evidence suggested that anorexia, vomiting, and a catabolic state are responsible for the poor growth associated with febrile illnesses in children [46]. However, other studies have reported no association of malaria with growth outcomes [47–50]. Similar to our study, most of these studies provided active malaria diagnosis and treatment, which may have attenuated the strength of the association.

In the studies cited above, different exposure and outcome measures were used, which may also explain the inconsistency in the findings. For example, we defined linear growth as change in LAZ and stunting. Change in LAZ indicates the growth rate between two time-points and therefore provides more information about linear growth faltering than stunting status assessed at one time-point [51, 52]. Therefore, it is possible that there is no association between malaria and linear growth (based on change in LAZ), hemoglobin or iron status, or the association is very weak. It is also possible that the association between malaria and these outcomes is only seen in children in the left end of the curve (i.e. LAZ < − 2 or hemoglobin < 110 g/L), hence the significant association of malaria with stunting and anemia but not change in LAZ or hemoglobin concentration. In contrast, diarrhea incidence was associated with an entire leftward shift in LAZ and gross motor scores in this population. A leftward shift in mean LAZ for a population is associated with increased risk of mortality [53].

The association of diarrhea with growth has been reported in previous studies [7–9]. Frequent diarrhea episodes result in persistent loss of nutrients necessary for growth through malabsorption, changes in gut microbiota, continuous immune system activation, increased metabolism and anorexia resulting in growth suppression. In our study, the magnitude of the significant associations of malaria and diarrhea with the outcomes were small, consistent with other studies [7, 42, 44, 54–56]. This could be partly attributable to the active surveillance and treatment provided to all children, or unmeasured confounding [44], or that perhaps other conditions such as chronic inflammation and environmental enteric dysfunction may be more important determinants of child growth in developing countries [57, 58]. Nevertheless, in a study combining nutrition intervention with treatment of malaria and diarrhea there was greater growth velocity, a 25% reduction in prevalence of stunting and improved developmental outcomes at age 18 months [4], suggesting that interventions that combine improved nutrition with control of infections may have significant impact.

## Conclusions

We conclude that in this population of children aged 6–18 mo living in a malaria-endemic setting, with active surveillance and early treatment, ‘presumed’ malaria is not associated with change in LAZ, hemoglobin or iron status, but could be associated with stunting and anemia. In this population, diarrhoea was more consistently associated with growth than was malaria or ARI. These findings may be different in contexts where there is no active case finding and treatment for malaria is not promptly administered.

## Abbreviations

ARI: Acute respiratory infection
CI: Confidence interval
Hb: Hemoglobin
HFIA: Household food insecurity access
IFA: Iron and folic acid
IMCI: Integrated Management of Childhood Illness
KDI: Kilifi Developmental Inventory
LAZ: Length-for-age z-scores
LNS: Lipid-based nutrient supplements
MMN: Multiple micronutrients
mRDT: Malaria Rapid Diagnostic Test
PSED: Profile of Social and Emotional Development
RR: Risk ratio
SD: Standard deviation
WAZ: Weight-for-age z-scores
WHO: World Health Organization
WLZ: Weight-for-length z-scores
ZPP: Zinc protoporphyrin
